# Can gadolinium contrast agents be replaced with saline for direct MR arthrography of the hip? A pilot study with arthroscopic comparison

**DOI:** 10.1007/s00330-023-09586-0

**Published:** 2023-04-12

**Authors:** Malin K. Meier, Moritz Wagner, Alexander Brunner, Till D. Lerch, Simon D. Steppacher, Peter Vavron, Ehrenfried Schmaranzer, Florian Schmaranzer

**Affiliations:** 1 Department of Orthopedic Surgery, Inselspital Bern, University Hospital, University of Bern, Freiburgstrasse, 3010 Bern, Switzerland; 2 Department of Orthopaedic Surgery, District Hospital St. Johann in Tirol, Bahnhofstrasse 14, 6380 St. Johann in Tirol, Austria; 3 Department of Diagnostic, Interventional and Pediatric Radiology, Inselspital, Bern University Hospital, University of Bern, Freiburgstrasse, 3010 Bern, Switzerland; 4 Department of Radiology, District Hospital St. Johann in Tirol, Bahnhofstrasse 14, 6380 St. Johann in Tirol, Austria

**Keywords:** MRI, Arthrography, Hip, Contrast agent, Arthroscopy

## Abstract

**Objective:**

To compare image quality and diagnostic performance of preoperative direct hip magnetic resonance arthrography (MRA) performed with gadolinium contrast agent and saline solution.

**Methods:**

IRB-approved retrospective study of 140 age and sex-matched symptomatic patients with femoroacetabular impingement, who either underwent intra-articular injection of 15–20 mL gadopentetate dimeglumine (GBCA), 2.0 mmol/L (“GBCA-MRA” group, *n* = 70), or 0.9% saline solution (“Saline-MRA” group, *n* = 70) for preoperative hip MRA and subsequent hip arthroscopy. 1.5 T hip MRA was performed including leg traction. Two readers assessed image quality using a 5-point Likert scale (1–5, excellent-poor), labrum and femoroacetabular cartilage lesions. Arthroscopic diagnosis was used to calculate diagnostic accuracy which was compared between groups with Fisher’s exact tests. Image quality was compared with the Mann–Whitney *U* tests.

**Results:**

Mean age was 33 years ± 9, 21% female patients. Image quality was excellent (GBCA-MRA mean range, 1.1–1.3 vs 1.1–1.2 points for Saline-MRA) and not different between groups (all *p* > 0.05) except for image contrast which was lower for Saline-MRA group (GBCA-MRA 1.1 ± 0.4 vs Saline-MRA 1.8 ± 0.5; *p* < 0.001). Accuracy was high for both groups for reader 1/reader 2 for labrum (GBCA-MRA 94%/ 96% versus Saline-MRA 96%/93%; *p* > 0.999/*p* = 0.904) and acetabular (GBCA-MRA 86%/ 83% versus Saline-MRA 89%/87%; *p* = 0.902/*p* = 0.901) and femoral cartilage lesions (GBCA-MRA 97%/ 99% versus Saline-MRA 97%/97%; both *p* > 0.999).

**Conclusion:**

Diagnostic accuracy and image quality of Saline-MRA and GBCA-MRA is high in assessing chondrolabral lesions underlining the potential role of non-gadolinium-based hip MRA.

**Key Points:**

• *Image quality of Saline-MRA and GBCA-MRA was excellent for labrum, acetabular and femoral cartilage, ligamentum teres, and the capsule (all p* > *0.18)*.

• *The overall image contrast was lower for Saline-MRA (Saline-MRA 1.8* ± *0.5 vs. GBCA-MRA 1.1* ± *0.4; p* < *0.001)*.

• *Diagnostic accuracy was high for Saline-MRA and GBCA-MRA for labrum (96% vs. 94%; p* > *0.999), acetabular cartilage damage (89% vs. 86%; p* = *0.902), femoral cartilage damage (97% vs. 97%; p* > *0.999), and extensive cartilage damage (97% vs. 93%; p* = *0.904)*.

**Supplementary Information:**

The online version contains supplementary material available at 10.1007/s00330-023-09586-0.

## Introduction

Direct magnetic resonance arthrography (MRA) of the hip is widely considered the diagnostic gold standard for the detection of intra-articular lesions in patients with hip deformities such as hip dysplasia or femoroacetabular impingement (FAI) [1, 2]. The desired effect of joint distension is usually achieved with intra-articular injection of highly diluted gadolinium-based contrast agents (GBCA). This procedure is reportedly safe, being associated with minimal postprocedural pain secondary to an inflammatory response to the contrast agent, and does rarely lead to allergic reactions [3]. There has been mounting evidence that GBCA administration at systemic dose levels leads to gadolinium deposition in the body [4]. Yet to date, GBCA deposition on brain magnetic resonance imaging (MRI) has not been reported in the two studies in which MRI subsequent to intra-articular injection of highly diluted GBCA was performed [5, 6]. There has been controversy surrounding potentially chondrotoxic effects related to the intra-articular injection of GBCA [7, 8]. Coupled with the costs of GBCA, patient concerns exist, which can lead to prolonged preprocedural informed consent consultations. Furthermore, reducing costs and patient concerns along with improving patient safety would yield medical and economic benefits alike. Previously, the suitability of alternative agents for direct MRA, mostly using physiologic saline solutions, has been evaluated for the shoulder [9–11] and the elbow [12]. Comparable diagnostic accuracy in detecting rotator cuff and glenoid labrum lesions was reported for saline and GBCA-MRA of the shoulder [13]. For MRA of the hip, a comparable image quality using hyaluronic acid as an alternative contrast agent was described [14].

Based on encouraging experience using saline as a contrast agent for direct shoulder MRA in our institution, saline MRA was included in our routine protocol for the hip joint.

The aim of this study was to compare image quality and diagnostic performance of direct MRA of the hip performed with GBCA and saline solution in patients undergoing hip arthroscopy.

## Material and methods

### Study design and participant inclusion

Following IRB approval with a waiver for informed consent, a retrospective study was performed at a primary hospital in Austria with a referral center for joint-preserving hip surgery. Inclusion criteria were patients with hip pain who had undergone direct hip MR arthrography with either gadopentetate dimeglumine (“GBCA-MRA” group) or saline solution (“Saline-MRA” group) as the intra-articular contrast agent and treated with subsequent hip arthroscopy. Hip pain diagnosis was established by a senior hip surgeon based on a history of symptoms for longer than 3 months and a positive impingement test, a positive apprehension test, or both, in the presence of osseous hip deformities [15, 16].

**Fig. 1:**
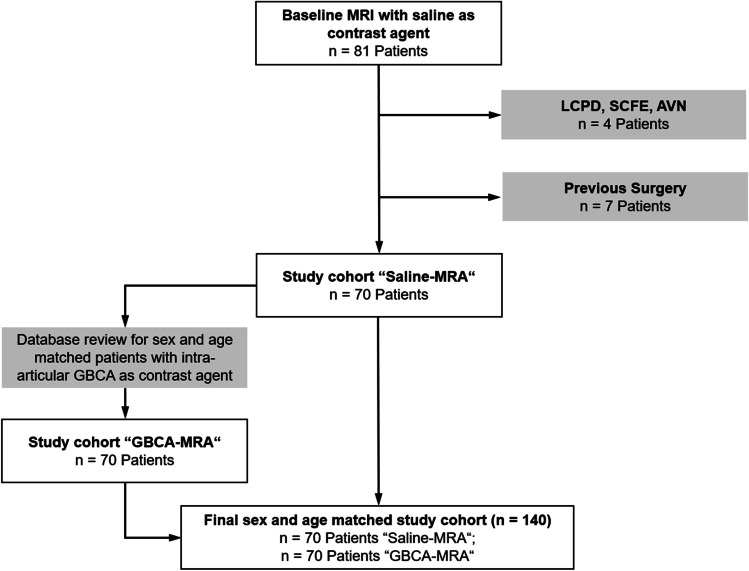
Flow diagram of patient inclusion

Beginning in January 2018, the institutional protocol was changed to hip MRA with saline solution following a positive experience from shoulder MRA. Moreover, the intention of this change was to reduce injection costs and alleviate patient anxiety from adverse events such as allergic reactions and GBCA deposition. This resulted in 81 patients undergoing preoperative Saline-MRA over 1.5 years (January 2018–June 2019) followed by subsequent hip arthroscopy. Exclusion criteria were age < 18 years, sequelae of Legg-Calvé-Perthes disease (LCPD), slipped capital femoral epiphysis (SCFE), and avascular necrosis of the hip (AVN). After the exclusion of 11 cases, this resulted in 70 patients for the Saline-MRA group. Subsequently, the institutional database was reviewed for sex and age (maximum ± 2 years difference) matched patients who did not meet any exclusion criteria and underwent direct MR arthrography with gadolinium as an intra-articular contrast agent between January 2014 and December 2017 followed by hip arthroscopy. This resulted in an overall study cohort of 140 patients (Fig. 1).

### Diagnostic imaging

AP pelvis and Dunn’s 45° views were obtained in a supine position [17]. Intra-articular injection of either 15–20 mL gadopentetate dimeglumine 2.0 mmol/L (GBCA-MRA group) or 0.9% NaCl solution (Saline-MRA group) was performed under fluoroscopic guidance using 1–2 mL iodinated contrast agent and additional injection of 2–5 mL local anesthetic. MRA was performed at the same 1.5 T scanner (Magnetom Aera, Siemens Healthineers) using a large flexible coil. Leg traction was applied using a method previously described and an MR-compatible traction device (TRACView; Menges Medical) [18–20] with a supporting plate for stabilization of the contralateral leg and a weight load adjusted to patients’ constitution (15 kg for patients < 60 kg, 18 kg for patients 60–80 kg, 23 kg for patients > 80 kg), which is connected to a cable via pulley to an ankle brace. The MR imaging protocol included multiplanar (i.e., coronal, sagittal, axial-oblique, and radial) PD-w TSE sequences without fat saturation and sequences of the pelvis and distal femoral condyles (Supplementary table 1). Post hoc cost analysis of the drugs needed for intra-articular injection was performed based on the current institutional purchasing prices: 20 mL gadopentetate dimeglumine 2.0 mmol/L (Magnevist; Bayer Healthcare; price: 46.54€), 50 mL NaCl solution 0.9% (0.9% NaCL Fresenius; Fresenius Kabi; price: 0.30€), 10 mL iopamidol 200 mg/mL (Iopamiro 200; Bracco; price: 0.96€), 10 mL ropivacaine hydrochloride 2 mg/mL (Ropinaest; Gebro Pharma; price: 0.93€). Accordingly, costs for GBCA-MRA were 48.43€ which were reduced by 46.24€ (− 95.5%) to 2.19€ for Saline-MRA.

### Image analysis

Analysis of imaging was performed independently by two blinded readers (radiologists with 12 years (E.S.) and 7 years (F.S.) of experience in hip imaging who were blinded to the operative records). One reader (E.S.) repeated the analysis after 6 months for evaluation of intra-rater reliability. The Tönnis grade of osteoarthritis, lateral center edge (LCE) angle according to Wiberg et al. [21], acetabular index, and signs for acetabular retroversion (crossover, posterior wall, ischial spine sign) [1, 17] were assessed on anteroposterior (AP) pelvis radiographs. Diagnosis of osseous deformities was made according to the 2020 Lisbon agreement on FAI imaging including measurement of alpha angles on radial images and assessment of femoral torsion according to Murphy et al. [1, 22–24].

MR image quality was assessed using a 5-point Likert scale ranging from 1 (excellent) to 5 (poor) for visibility of labrum, acetabular cartilage, femoral cartilage, ligamentum teres, and capsule [14]. Additionally, overall image contrast was assessed using the Likert scale. The presence/absence of joint distraction was recorded if a layer of contrast was visible between the femoral and acetabular cartilage layers on coronal PD-w TSE images obtained with traction [18].

**Fig. 2:**
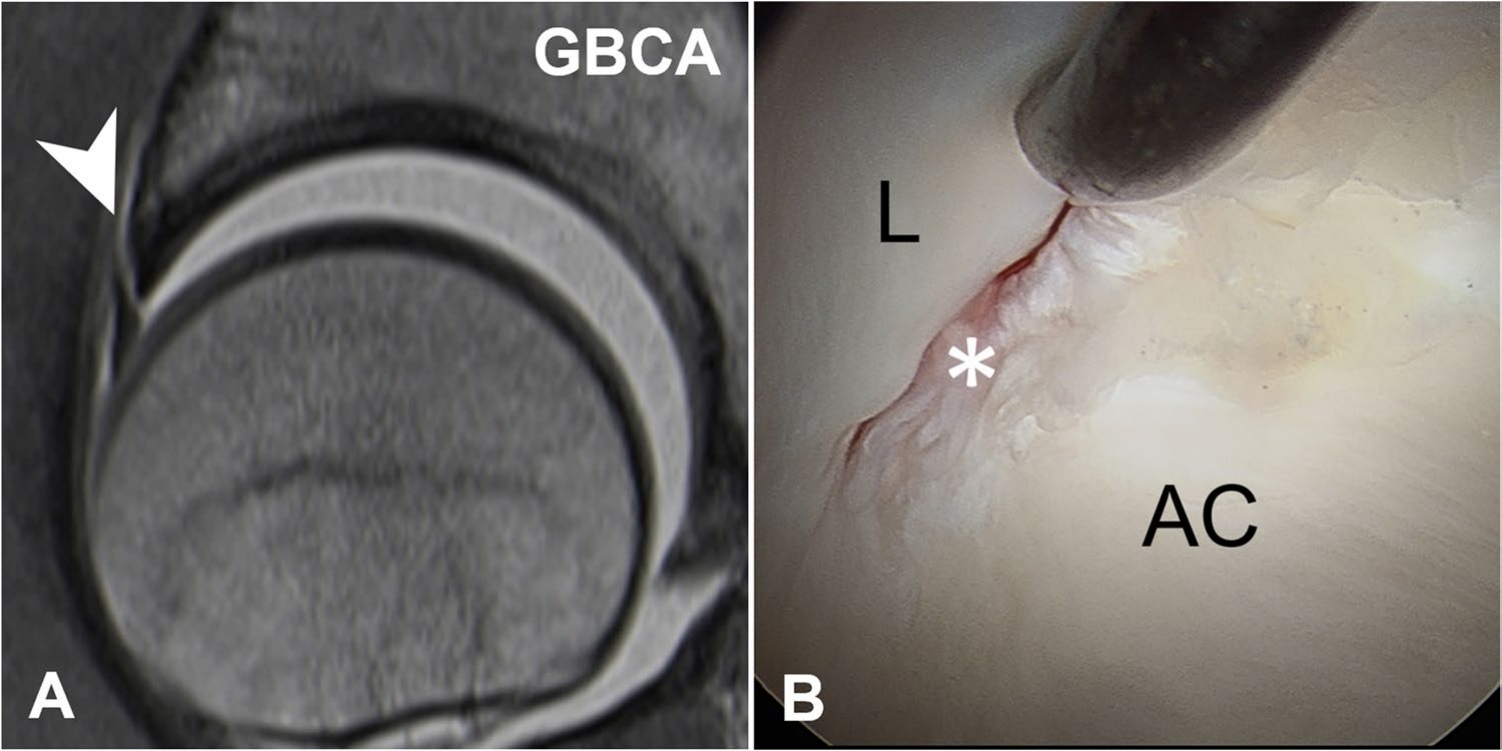
**A** 40-year-old patient with preoperative GBCA-MR arthrogram showing an anterior labrum lesion (arrowhead) in the sagittal PD-w TSE image, (**B**) which could be confirmed arthroscopically (asterisk). Surgical images—L labrum, AC acetabular cartilage

**Fig. 3:**
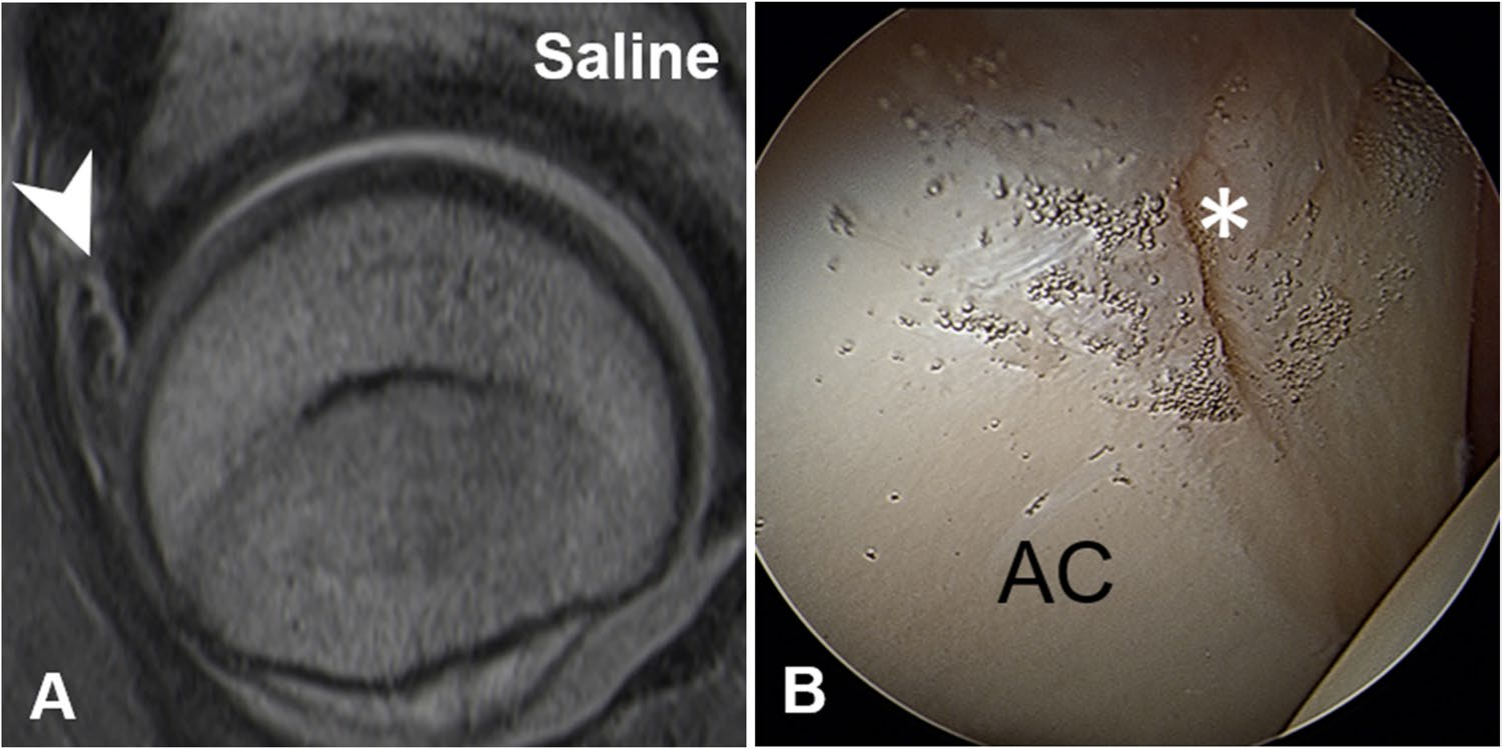
**A** 20-year-old patient with preoperative Saline-MR arthrogram with slightly lower image quality due to less bright joint fluid compared to Fig. 2. Despite that, an anterior labrum lesion (arrowhead) is clearly depicted in the sagittal PD-w TSE image, (**B**) which was confirmed arthoscopically (asterisk). Surgical images—AC acetabular cartilage

**Fig. 4:**
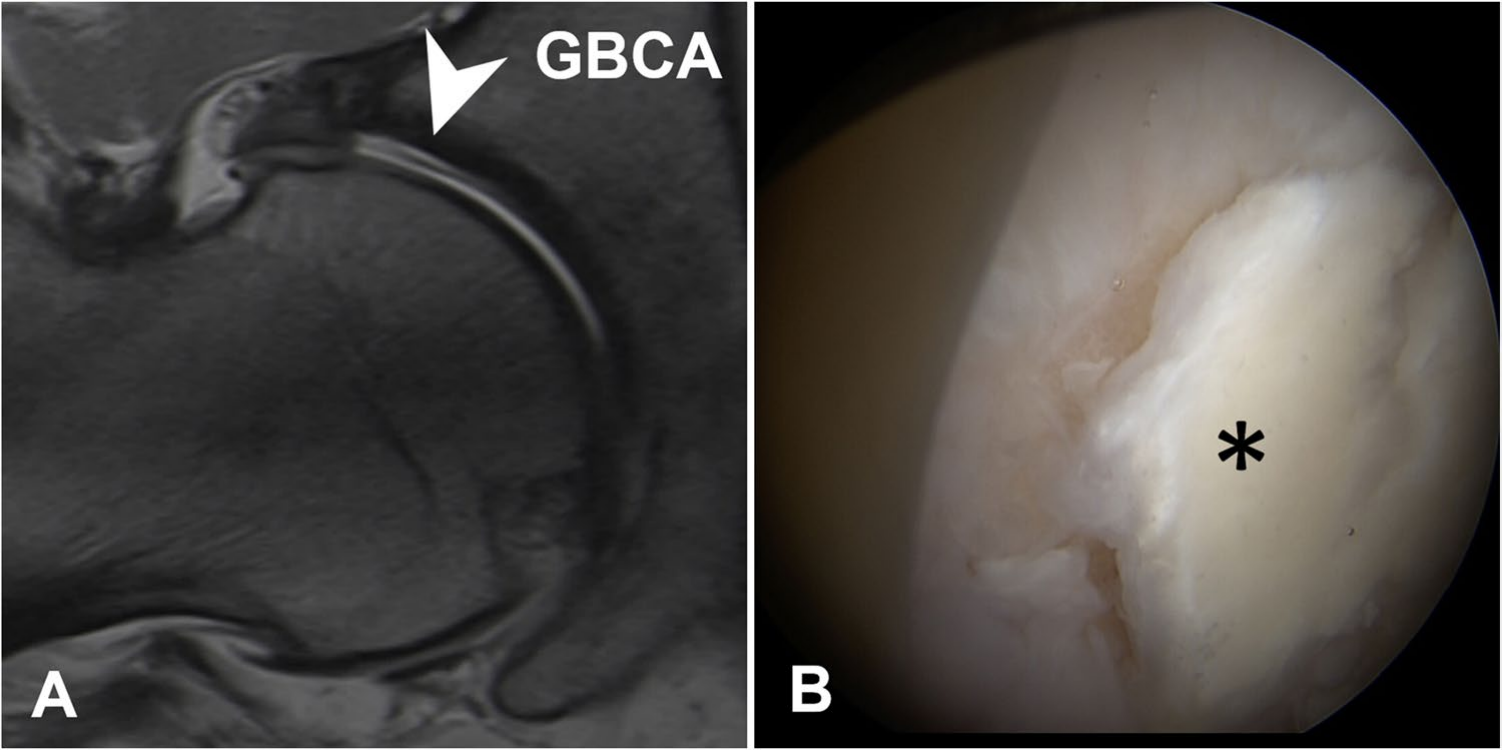
**A** 36-year-old patient with preoperative GBCA-MR arthrogram showing an acetabular cartilage delamination in the radial PD-w TSE image (arrowhead), (**B**) which was confirmed arthroscopically (asterisk)

**Fig. 5:**
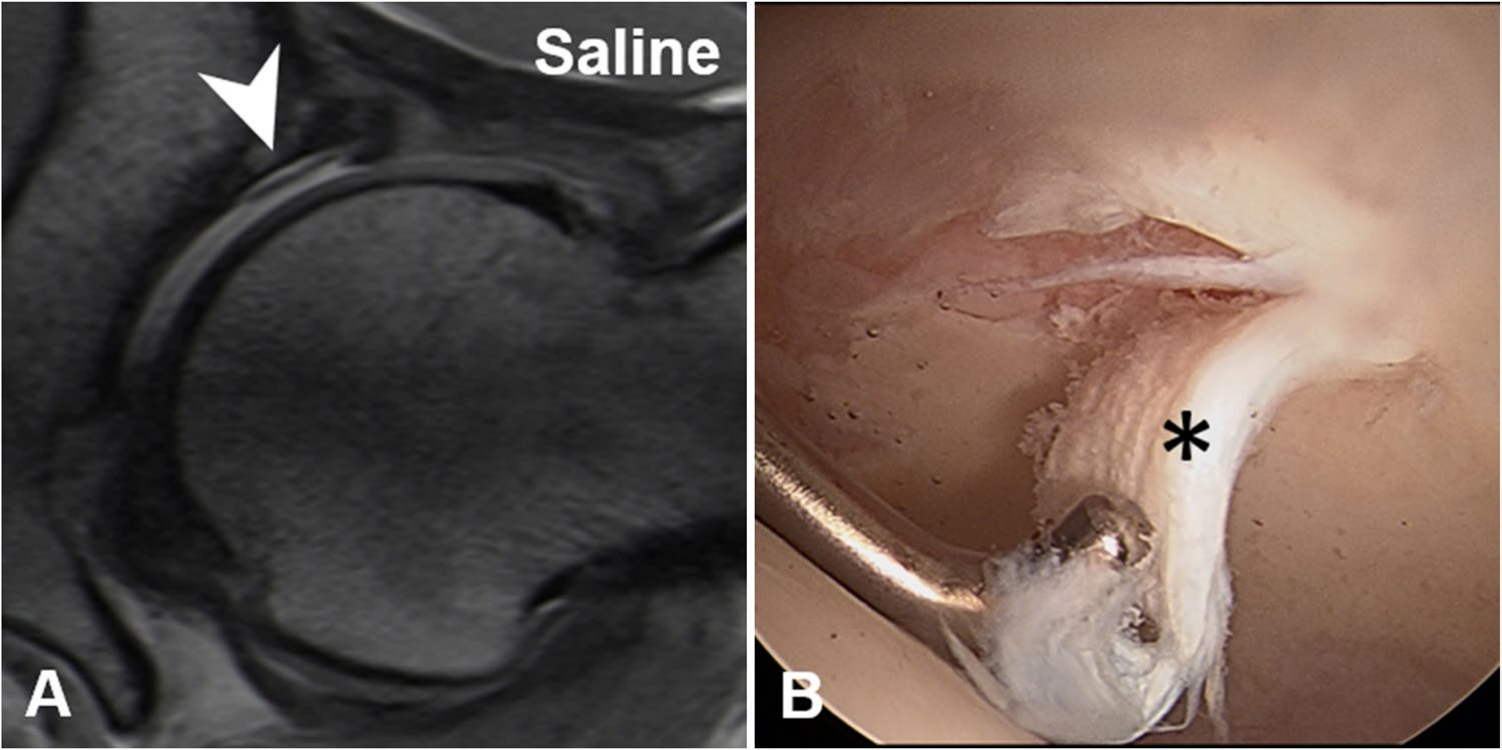
**A** 27-year-old patient with preoperative Saline-MR arthrogram showing an acetabular cartilage delamination (arrowhead) in the radial PD-w TSE image, (**B**) which was confirmed arthroscopically (asterisk)

**Fig. 6:**
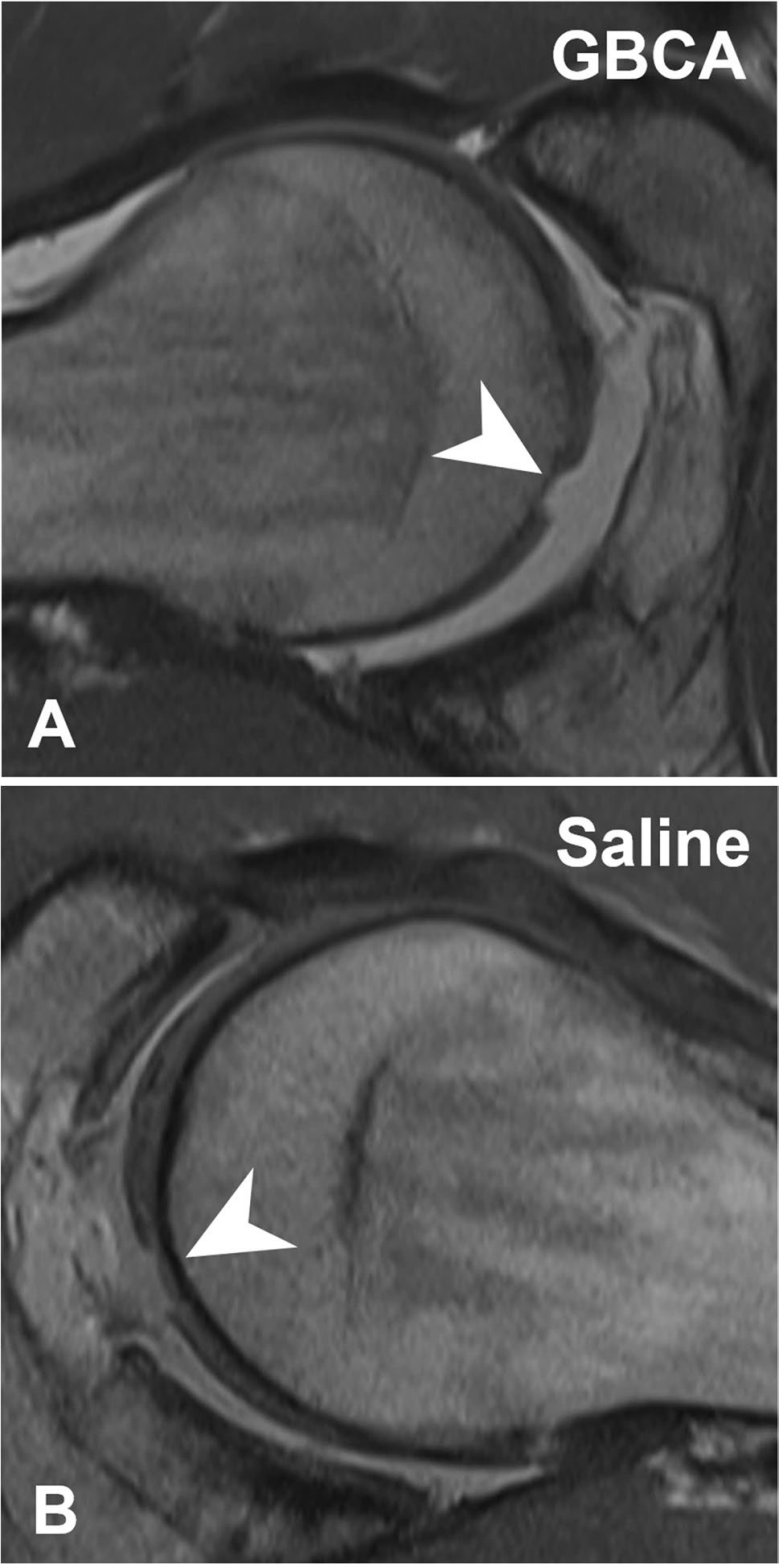
**A** Right hip of a 35-year-old patient with preoperative GBCA-MR arthrogram. **B** Left hip of the same patient with Saline-MR arthrogram. Both axial-oblique images show femoral cartilage lesion (arrowheads) with similar overall image quality

MR images were evaluated for labrum and cartilage lesions (acetabular and femoral) to assess diagnostic performance. Labrum lesions were graded as intersubstance (fluid signal extending between the acetabular rim and the labral base) and intra-substance tears (fluid signal extending into the labral substance) [25] (Figs. 2 and 3). Acetabular and femoral cartilage damage was graded as delamination, thinning, or defect [25] (Figs. 4, 5, and 6). The presence of extensive cartilage damage > 2 h on the clock face (i.e., 60°) was recorded as well because it represents a negative predictor for the success of FAI surgery [26–28]. The location of the chondrolabral damage was recorded on MRA and intra-operatively with the standard clock-face system dividing the joint into 12 clock-face positions [1]. Arthroscopic diagnosis of the chondrolabral damage served as the reference for the calculation of diagnostic performance.

### Hip arthroscopy

Hip arthroscopy was performed in a supine position by the same orthopedic surgeon with 12 years of experience. Chondrolabral damage was photo-documented with the arthroscope and described in the surgical report. For comparison with imaging findings, surgical records were reviewed and a diagnosis of the chondrolabral damage was extracted by an orthopedic surgeon. Labrum damage was graded as intersubstance and intra-substance tears [25]. Acetabular and femoral cartilage damage was graded as delamination, thinning, or defect [25].

### Statistical analysis

Data are displayed as mean ± standard deviation with 95% confidence intervals.

Normal distribution of continuous data was confirmed using the Kolmogorov–Smirnov test. Likert scores were compared between groups via the Mann–Whitney *U* test. Diagnostic performance was assessed with sensitivity, specificity, accuracy, and positive and negative predictive value and compared between groups using Fisher’s exact test for both readers. Agreement between MRA and arthroscopic gradings of the chondrolabral damage was calculated with Cohen’s kappa (*ĸ*) and 95% confidence intervals. Interrater and intra-rater reliability was assessed with Cohen’s kappa (*ĸ*) and 95% confidence intervals. Interpretation of inter- and intrarater agreement was performed as follows: *ĸ* values ≤ 0 as indicating no agreement, 0.01–0.20 none to slight, 0.21–0.40 fair, 0.41–0.60 moderate, 0.61–0.80 substantial, and 0.81–1.00 almost perfect agreement [29].

A type I error rate of 5% was used to determine statistical significance. Statistical analysis was performed with GraphPad Prism (Version 9.1, GraphPad Software).

## Results

### Patient characteristics

The database was reviewed for patients undergoing Saline-MRA and consecutive hip arthroscopy over a 1.5 years period (January 2018–June 2019). Out of 81 patients, 11 patients were excluded (7 with previous surgery, 1 with SCFE, 1 with LCPD, and 2 with AVN (Fig. 1). These 70 hips were age- and sex-matched with 70 patients undergoing MRA with GBCA Mean age did not differ between the GBCA-MRA (mean age of 33 ± 9 years) and the Saline-MRA (mean age of 32.5 ± 10 years; *p* = 0.517). Both groups had 21% (15/70) female patients (*p* > 0.999). Preoperative osseous hip deformities did not differ between both groups (all *p* > 0.05) (Supplementary table 2). Joint distraction was achieved for both groups (mean ± SD; joint distraction GBCA-MRA 4.3 ± 2.1 mm, Saline-MRA 4.7 ± 2.1 mm; *p* = 0.319). The time interval between MRA and hip arthroscopy did not differ between both groups (mean ± SD; GBCA-MRA 3.9 ± 3.1 months vs. Saline-MRA 4.7 ± 3.9 months; *p* = 0.102).

### Image quality

Image quality (Likert scale) was excellent for the Saline-MRA group with a range of 1.1 ± 0.3 for the ligamentum teres to 1.2 ± 0.4 for the labrum and acetabular cartilage and the GBCA-MRA group (*p* > 0.05) with a range of 1.1 ± 0.3 for femoral cartilage to 1.3 ± 0.5 for the capsule (Table 1). Overall image contrast was significantly higher in the GBCA-MRA group (1.1 ± 0.4) compared to the Saline-MRA group (1.8 ± 0.5); *p* < 0.001.

**Table 1:** Image quality

Likert scale	Reader 1			Reader 2		
	GBCA-MRA	Saline-MRA	*p* value	GBCA-MRA	Saline-MRA	*p* value
Labrum	1.2 ± 0.5	1.2 ± 0.4	0.198	1.2 ± 0.5	1.3 ± 0.4	0.646
Acetabular cartilage	1.3 ± 0.6	1.2 ± 0.4	0.853	1.2 ± 0.5	1.2 ± 0.4	0.952
Femoral cartilage	1.1 ± 0.3	1.1 ± 0.3	0.882	1.2 ± 0.4	1.1 ± 0.3	0.419
Ligamentum teres	1.2 ± 0.4	1.1 ± 0.3	0.183	1.1 ± 0.4	1.1 ± 0.3	0.216
Capsule	1.3 ± 0.5	1.2 ± 0.5	0.782	1.2 ± 0.4	1.3 ± 0.5	0.400
Overall contrast	1.1 ± 0.4	1.8 ± 0.5	< 0.001	1.1 ± 0.3	1.9 ± 0.4	0.019

Mean ± SD., Mann–Whitney *U* test was used for comparison of Likert scale 1 (excellent) to 5 (poor)

The results of reader 2 can be found in Table 1.

### Diagnostic performance

Accuracy was high for both groups for labrum (GBCA-MRA 94% [95% CI: 83–97] versus Saline-MRA 96% [88–99]; *p* > 0.999), acetabular cartilage (GBCA-MRA 86% [75–93] versus Saline-MRA 89% [78–95]; *p* = 0.902), femoral cartilage (GBCA-MRA and Saline-MRA both 97% [90–100]; *p* > 0.999), and extensive cartilage damage (GBCA-MRA 93% [84–98] versus Saline-MRA 97% [90–100]; *p* = 0.904; Table 2). For the diagnostic performance of reader 2, see Table 3.

**Table 2:** Diagnostic performance of GBCA-MRA and Saline-MRA of reader 1

	Labrum			Acetabular cartilage			Femoral cartilage			Extensive cartilage damage > 2 h (60°)		
	GBCA	Saline-MRA	*p* value	GBCA-MRA	Saline-MRA	*p* value	GBCA-MRA	Saline-MRA	*p* value	GBCA-MRA	Saline-MRA	*p* value
True positive (no. of hips)	64/70	66/70	-	46/70	44/70	-	9/70	9/70	-	13/70	13/70	-
True negative (no. of hips)	2/70	1/70	-	14/70	18/70	-	59/70	59/70	-	52/70	55/70	-
False positive (no of hips)	4/70	2/70	-	2/70	1/70	-	2/70	1/70	-	2/70	2/70	-
False negative (no. of hips)	0/70	1/70	-	8/70	7/70	-	0/70	1/70	-	3/70	0/70	-
Sensitivity (%)	100 (94–100)	99 (92–100)	> 0.999	85 (73–92)	86 (74–93)	> 0.999	100 (70–100)	90 (60–99)	> 0.999	81 (57–93)	100 (77–100)	0.790
Specificity (%)	33 (6–70)	33 (2–88)	> 0.999	88 (64–98)	95 (75–100)	> 0.999	97 (89–99)	98 (91–100)	> 0.999	96 (87–99)	97 (88–99)	> 0.999
PPV (%)	94 (86–98)	97 (90–99)	0.903	96 (86–99)	98 (88–100)	> 0.999	82 (52–97)	90 (60–99)	> 0.999	87 (62–98)	87 (62–98)	> 0.999
NPV (%)	100 (18–100)	50 (3–97)	> 0.999	64 (43–80)	72 (52–86)	0.822	100 (94–100)	98 (91–100)	> 0.999	95 (85–99)	100 (94–100)	0.892
Accuracy (%)	94 (83–97)	96 (88–99)	> 0.999	86 (75–93)	89 (78–95)	0.902	97 (90–100)	97 (90–100)	> 0.999	93 (84–98)	97 (90–100)	0.904
Grading agreement MRI versus surgery (*ĸ*)	0.58 (0.41–0.74)	0.69 (0.53–0.86)	-	0.63 (0.47–0.79)	0.66 (0.51–0.82)	-	0.86 (0.68–1.00)	0.89 (0.74–1.00)	-	0.79 (0.62–0.97)	0.91 (0.79–1.00)	-

Diagnostic performance was compared with Fisher’s exact test. Agreement between MRI and surgical lesion grades was assessed with Cohen’s *κ*

**Table 3:** Diagnostic performance of GBCA-MRA and Saline-MRA of reader 2

	Labrum			Acetabular cartilage			Femoral cartilage			Extensive cartilage damage > 2 h (60°)		
	GBCA-MRA	Saline-MRA	*p* value	GBCA-MRA	Saline-MRA	*p* value	GBCA-MRA	Saline-MRA	*p* value	GBCA-MRA	Saline-MRA	*p* value
True positive (no. of hips)	64/70	64/70	-	46/70	46/70	-	9/70	8/70	-	14/70	12/70	-
True negative (no. of hips)	3/70	1/70	-	12/70	15/70	-	60/70	60/70	-	54/70	55/70	-
False positive (no of hips)	3/70	2/70	-	4/70	4/70	-	1/70	0/70	-	0/70	2/70	-
False negative (no. of hips)	0/70	3/70	-	8/70	5/70	-	0/70	2/70	-	2/70	1/70	-
Sensitivity (%)	100 (94–100)	96 (88–99)	> 0.999	85 (73–92)	90 (79–96)	0.887	100 (71–100)	80 (49–96)	> 0.999	88 (64–98)	92 (67–100)	> 0.999
Specificity (%)	50 (12–88)	33 (2–88)	0.571	75 (50–90)	79 (57–92)	> 0.999	98 (91–100)	100 (94–100)	> 0.999	100 (93–100)	97 (88–99)	> 0.999
PPV (%)	96 (91–98)	97 (90–100)	> 0.999	92 (81–97)	92 (81–97)	> 0.999	90 (60–100)	100 (68–100)	> 0.999	100 (79–100)	86 (60–98)	0.793
NPV (%)	100 (18–100)	25 (1–70)	> 0.999	60 (39–78)	75 (53–89)	0.804	100 (94–100)	97 (89–99)	> 0.999	96 (88–99)	98 (91–100)	> 0.999
Accuracy (%)	96 (88–99)	93 (84–98)	0.904	83 (72–91)	87 (77–94)	0.901	99 (92–100)	97 (90–100)	> 0.999	97 (88–99)	96 (88–99)	> 0.999
Grading agreement MRI versus surgery (*ĸ*)	0.67 (0.51–0.83)	0.56 (0.38–0.74)	-	0.45 (0.26–0.64)	0.60 (0.43–0.77)	-	0.88 (0.73–1.00)	0.89 (0.71–1.00)	-	0.92 (0.80–1.00)	0.86 (0.71–1.00)	-

Diagnostic performance was compared with Fisher’s exact test. Agreement between MRI and surgical lesion grades was assessed with Cohen’s *κ*

### Interrater and intra-rater reliability

Interrater reliability (*ĸ*) was substantial to almost perfect for diagnosing acetabular (0.66 [0.47–0.85]) and femoral cartilage damage (0.94 [0.84–1.00]) with GBCA-MRA and Saline-MRA, respectively (Table 4). For the labrum, interrater reliability was substantial for GBCA-MRA (0.79 [0.40–1.00]) and fair for Saline-MRA (0.31 [− 0.19–0.80]) (Table 4). Intra-rater reliability (*κ*) ranged from moderate for labrum (0.55 [0.11–0.99]) to almost perfect for femoral cartilage lesions (0.83 [0.65–1]) for both groups (Table 5).

**Table 4:** Interobserver agreement

Parameter	GBCA-MRA *κ* value	95% CI	Saline-MRA *κ* value	95% CI
Intra-articular lesion				
Labrum	0.79	0.40–1.00	0.31	–0.19–0.80
Acetabular cartilage	0.66	0.47–0.85	0.77	0.61–0.93
Femoral cartilage	0.94	0.84–1.00	0.87	0.70–1.00
Extensive cartilage damage	0.78	0.60–0.80	0.96	0.79–1.00
Likert				
Labrum	0.60	0.63–0.84	0.55	0.33–0.78
Acetabular cartilage	0.84	0.69–0.98	0.66	0.44–0.88
Femoral cartilage	0.57	0.28–0.85	0.54	0.27–0.88
Ligamentum teres	0.40	0.12–0.68	0.41	0.06–0.76
Capsule	0.56	0.34–0.77	0.54	0.31–0.76
Overall contrast	0.64	0.33–0.96	0.49	0.25–0.73

Kappa statistics were performed using Cohen’s *κ*. All *p* values < 0.01

**Table 5:** Intra-observer agreement

Parameter	GBCA-MRA *κ* value	95% CI	Saline-MRA *κ* value	95% CI
Intra-articular lesion				
Labrum	0.55	0.11–0.99	0.65	0.20–1.00
Acetabular cartilage	0.71	0.54–0.89	0.73	0.56–0.90
Femoral cartilage	0.83	0.65–1.00	0.77	0.55–0.99
Extensive cartilage damage	0.81	0.64–0.99	0.82	0.66–0.99
Likert				
Labrum	0.89	0.73–1.00	0.88	0.75–1.00
Acetabular cartilage	0.79	0.62–0.96	0.92	0.80– 1.00
Femoral cartilage	0.79	0.50–1.00	0.78	0.54–1.00
Ligamentum teres	0.84	0.67–1.00	0.82	0.57–1.00
Capsule	0.79	0.62–0.95	0.79	0.61–0.97
Overall contrast	0.93	0.78–1.00	0.77	0.25–1.00

Kappa statistics were performed using Cohen’s *κ*. All *p* values < 0.001

## Discussion

Although severe adverse events related to intra-articular injection of gadolinium-based contrast agents for direct MR arthrography are extremely rare [3], the use of a saline solution could bypass patient concerns and reduce costs. Therefore, we compared image quality and diagnostic performance in the detection of intra-articular lesions of the hip between Saline- and GBCA-MRA (Figs. 5 and 6).

Image quality was excellent for Saline-MRA and GBCA-MRA for the assessment of the labrum, acetabular and femoral cartilage, hip capsule, and ligamentum teres. Overall image contrast was lower (GBCA-MRA 1.1 ± 0.4 vs. Saline-MRA 1.8 ± 0.5 Likert scale; *p* < 0.001), since the intra-articular fluid was less bright in Saline-MRA.

In addition, interobserver agreement for labrum lesions for Saline-MRA was only fair (*ĸ* = 0.31), while substantial (*ĸ* = 0.79) for GBCA-MRA. The higher variability between readers for the detection of labrum tears based on Saline-MRA may be explained by the potentially less intense signal interposition within or at the labral base as the intra-articular fluid was less bright compared to GBCA-MRA. This may be further complicated by the fact that labrum lesions reportedly present as intermediate signal alterations in up to 28% of patients on GBCA-MRA and thus can be confused with mucoid degeneration [30]. This may explain the increased variability between two different readers in diagnosing labrum lesions on Saline-MRA as opposed to the assessment of femoral and acetabular cartilage lesions that showed at least substantial interrater reliability for both techniques. Furthermore, we cannot rule out that differences in reader experience may have contributed to this finding. Literature about non-gadolinium-based contrast agents for direct hip MRA is sparse. One study compared the image quality of hip MR arthrography including fat-saturated fluid-sensitive images with hyaluronic acid against GBCA as an intra-articular contrast agent and reported comparable results for image contrast for cartilage, labrum, and hip capsule. Yet, intra-articular lesions were not compared to surgical findings [14].

In a previous study, Tiegs-Heiden et al. [31] tried to simulate hip MRA with saline by evaluating T2-weighted sequences thereby eliminating the GBCA effect versus assessment of T1-weighted images in 75 patients undergoing GBCA-MRA followed by hip arthroscopy [31]. They reported no inferiority (difference in the area under the curve—0.004; *p* = 0.90 for the labrum and 0.011; *p* = 0.79 for acetabular cartilage lesions) for analysis of T2-weighted sequences only in the detection of chondrolabral lesions. They proposed a direct comparison between GBCA- versus Saline-MRA in future studies since this comparison had not been possible in their study [31]. In contrast to the aforementioned studies [14, 31], we performed a comparison between age- and sex-matched patients undergoing either GBCA- or Saline-MRA of the hip followed by hip arthroscopy. Diagnostic performance was high for Saline-MRA and GBCA-MRA for the detection of labrum lesions, femoroacetabular damage, and extensive cartilage damage (all *p* > 0.05). For Saline-MRA, sensitivity was 99% for labrum, 86% for acetabular cartilage, and 90% for femoral cartilage damage. This is comparable to a previous pilot study [19] on GBCA-MRA of the hip performed under leg traction, which reported a sensitivity of 93% for labrum, 88% for acetabular cartilage, and 86% for femoral cartilage damage [19]. Using traction during MRA of the hip to improve visualization of intra-articular lesions enabled detection rates higher than previously reported in the literature. Specificity in diagnosing labrum lesions was low for either technique (each 33%), which is most likely related to the over-representation of labrum lesions as patients with lesions are more likely to undergo surgery [31, 32]. A meta-analysis of 12 studies including 828 cases reported a pooled sensitivity of 91% and 86% for the detection of labrum lesions with conventional MR arthrography and non-contrast MRI, respectively. Furthermore, a pooled sensitivity of 75% for chondral lesions detected with MR arthrography and 76% for non-contrast MRI was reported [32].

Our results showed that extensive cartilage damage can be detected with high accuracy regardless of the injection of GBCA or saline (93% for GBCA-MRA vs. 97% for Saline-MRA), which is an important predictor for the success of joint-preserving surgery at short and long term [26, 27].

There are several limitations of this retrospective study. First, we cannot directly extrapolate our findings to conventional MR arthrography of the hip, since the application of traction is part of our institutional routine, and imaging with and without traction was not feasible in a routine clinical setting. This should be the subject of future studies. Second, an a priori sample size calculation was not feasible due to a lack of data reporting on the diagnostic performance of non-GBCA-MRA of the hip. Third, no optimization of image contrast for Saline-MRA was performed. Although T1-w images with fat saturation are commonly performed, PD-w TSE imaging without fat saturation is preferred in our institution as it enables the detection of osseous changes and yields good contrast of the cartilage and labrum alike [33]. Although diagnostic accuracy was comparable to a previous study in which T1-w-based traction MRA of the hip was performed [19], our results need to be confirmed on different pulse sequences including T1-w TSE imaging. The application of fat saturation and the use of longer echo times to achieve intermediate-weighted imaging can potentially improve the contrast of the injected saline [33]. In addition, the use of next-generation MRI scanners and hardware may further yield improved image quality. Fourth, even though the radiologists were blinded regarding the use of GBCA/saline, differences in overall image quality may have introduced a bias during the image analysis. Finally, we could only perform a post hoc cost analysis which may differ among countries and healthcare systems and could not compare patient anxiety/adverse events due to the retrospective study design.

To conclude, the image quality and diagnostic accuracy of Saline-MRA and GBCA-MRA in assessing chondrolabral lesions are high. This underlines the potential of omitting GBCA in the future and reducing costs.

## Supplementary Information

Below is the link to the electronic supplementary material.

Supplementary file1 (DOCX 20 KB)

## Abbreviations

AP: Anteroposterior
AVN: Avascular necrosis of the femoral head
FAI: Femoroacetabular impingement
GBCA: Gadolinium-based contrast agent
IRB: Institutional review board
LCE: Lateral center edge
LCPD: Legg-Calvé-Perthes disease
MRA: Magnetic resonance arthrography
MRI: Magnetic resonance imaging
PD-w: Proton-density-weighted
PPV: Positive predictive value
NPV: Negative predictive value
SCFE: Slipped capital femoral epiphysis
SD: Standard deviation
TSE: Turbo spin echo

## Declarations

### Guarantor

The scientific guarantor of this publication is Florian Schmaranzer.

### Statistics and biometry

No complex statistical methods were necessary for this paper.

### Methodology

• retrospective

• diagnostic study

• performed at one institution
