# Correction: Can gadolinium contrast agents be replaced with saline for direct MR arthrography of the hip? A pilot study with arthroscopic comparison

**DOI:** 10.1007/s00330-023-09844-1

**Published:** 2023-07-04

**Authors:** Malin K. Meier, Moritz Wagner, Alexander Brunner, Till D. Lerch, Simon D. Steppacher, Peter Vavron, Ehrenfried Schmaranzer, Florian Schmaranzer

**Affiliations:** 1 Department of Orthopedic Surgery, Inselspital Bern, University Hospital, University of Bern, Freiburgstrasse, 3010 Bern, Switzerland; 2 Department of Orthopaedic Surgery, District Hospital St. Johann in Tirol, Bahnhofstrasse 14, 6380 St. Johann in Tirol, Austria; 3 Department of Diagnostic, Interventional and Pediatric Radiology, Inselspital, Bern University Hospital, University of Bern, Freiburgstrasse, 3010 Bern, Switzerland; 4 Department of Radiology, District Hospital St. Johann in Tirol, Bahnhofstrasse 14, 6380 St. Johann in Tirol, Austria

**Correction: European Radiology**

**https://doi.org/10.1007/s00330-023-09586-0**

In this article the wrong figure appeared as Fig. 6B; the figure should have appeared as shown below.
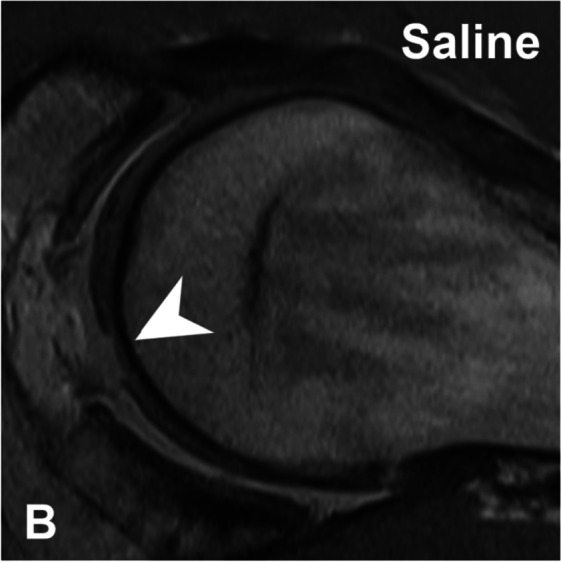


The original article has been corrected.

