# Supplementary material for: Can gadolinium contrast agents be replaced with saline for direct MR arthrography of the hip? A pilot study with arthroscopic comparison
Source: Eur Radiol. 2023 Apr 12;33(9):6369–80. doi: 10.1007/s00330-023-09586-0 (PMC10415454; doi:10.1007/s00330-023-09586-0)
Supplement: Supplementary file 1 — Supplementary file1 (DOCX 20 KB) [file 330_2023_9586_MOESM1_ESM.docx]

**Supplementary information**

**Supplementary table 1**. MR Sequence protocol.

| Sequence | Repetition  Time (ms) | Echo  Time (ms) | Matrix | FOV (mm) | Flip  Angle | Slice  Thickness (mm) | Bandwidth  (Hz/Px) | Image orientation | Traction |
| --- | --- | --- | --- | --- | --- | --- | --- | --- | --- |
| PD-w turbo spin echo | 2460 | 13 | 512 x 512 | 180 | 150 | 3 | 130 | coronal/ sagittal/ axial-oblique/ radial | yes |
| T1-w VIBE DIXON | 6.7 | 2.4/ 4.8 | 320 x 320 | 380 | 10 | 3 | 470 | axial pelvis and knee | no |

PD-w images were acquired without fat-saturation. Imaging under traction lasted 14-16 minutes. VIBE = volume interpolated breath-hold-examination.

**Supplementary table 2.** Demographic characteristics and radiographic parameters.

| Patient characteristics | GBCA-MRA | Saline-MRA | p-value |
| --- | --- | --- | --- |
| Demographic characteristics |  |  |  |
| Number of patients (n) | 70 | 70 | >0.999 |
| Age (y) | 33 ± 9 | 32.5 ± 10 | 0.517 |
| Female patients (%) | 21 (15/70) | 21 (15/70) | >0.999 |
| Time between MRI and surgery (months) | 3.9 ± 3.1 | 4.7 ± 3.9 | 0.102 |
| Radiographic parameters |  |  |  |
| Tönnis grade ≥1 (%) | 51 (36/70) | 44 (31/70) | 0.500 |
| Severe Retroversion (%) | 9 (6/70) | 19 (13/70) | 0.137 |
| Overcoverage (%) | 9 (6/70) | 21 (15/70) | 0.056 |
| Cam Deformity (%) | 89 (62/70) | 86 (60/70) | 0.801 |
| High femoral version (%) | 63 (44/70) | 53 (37/70) | 0.304 |
| Femoral Retroversion (%) | 0 (1/70) | 0 (3/70) | 0.620 |

Cam deformity defined as maximum alpha angle > 60° on radial images, acetabular overcoverage defined with a LCE > 35°, and acetabular retroversion defined as presence of three retroversion signs. High femoral torsion was defined as > 25° and low femoral torsion as < 10°. Continuous data was compared with unpaired t-test, categorial data was compared with Fisher’s exact test.
